# Interrow cover crops in a semi-arid vineyard increase plant beneficial functional potential of the soil microbiome, both in vine rows and interrows, a benefit that increases with cover crop duration

**DOI:** 10.1186/s40793-025-00726-1

**Published:** 2025-06-11

**Authors:** Fernando Igne Rocha, Jean Carlos Rodriguez-Ramos, Margaret Fernando, Lauren Hale

**Affiliations:** San Joaquin Valley Agricultural Sciences Center, USDA, Agricultural Research Service, 9611 South Riverbend Avenue, Parlier, CA 93648-9757 USA

**Keywords:** Cover crops, Vineyard soil health, Microbial networks, Mediterranean agriculture, *Phacelia tanacetifolia*, *Secale cereale*

## Abstract

**Background:**

Cover crops are recognized for enhancing soil health and providing agroecosystem services, but are not widely adopted, particularly in water-limited regions. In Mediterranean vineyards, where water scarcity and soil degradation challenge productivity, interrow, cool-season cover cropping offers a promising strategy to improve microbial-mediated soil functions. However, the temporal and spatial effects of cover crops on vineyard soil microbiomes and soil health metrics remain poorly understood. This study evaluated the impacts of a California native (phacelia, *Phacelia tanacetifolia*) and introduced (rye, *Secale cereale* L.) plant species as interrow cover crops on soil properties in interrow and vine row soils across three years.

**Results:**

The study revealed distinct temporal and spatial dynamics in soil microbiomes elicited by the cover crop treatments. By the third year, phacelia exhibited the highest microbial biomass, fungal-to-bacterial ratios, and microbial network complexity. Interrow soils showed stronger responses to cover cropping, including enhanced microbial biomass and differentiation between treatments, while vine row soils demonstrated subtler but significant shifts in microbial metrics. Functional predictions indicated that cover crops reduced fungal pathogen prevalence and supported nutrient cycling processes. Deterministic processes driven by environmental selection became dominant under both treatments, promoting microbial resilience. Random Forest analysis identified NO_3_^−^ as a key driver of microbial differentiation, with phacelia fostering communities reliant on labile organic inputs.

**Conclusions:**

This study highlights a crucial benefit of interrow cover crops in improving soil health and enhancing microbial-mediated ecosystem functions in adjacent vine row soils, even after cover crop termination. Long-term application of cover crops offers a sustainable approach to building resilient agroecosystems in water-scarce environments, with implications for sustainable viticulture practices.

**Supplementary Information:**

The online version contains supplementary material available at 10.1186/s40793-025-00726-1.

## Highlights

Phacelia cover increases microbial network complexity and resilience in vineyard soils.

Rye cover boosts microbial biomass despite reduced nitrogen availability.

Arbuscular mycorrhizal fungi abundance rises under cover crops, enhancing soil health.

Microbiome structuring is shaped by cover crops with intensified effects in the interrow.

Soil microbiomes become increasingly distinct with increased duration of cover cropping management.

**Supplementary Information:**

The online version contains supplementary material available at 10.1186/s40793-025-00726-1.

## Introduction

Modern agricultural systems face the challenge of maintaining productivity while mitigating the impacts of soil degradation, water scarcity, and declining ecosystem services associated with monocultures [1]. Cover crops provide multifaceted benefits, including improving soil structure, reducing topsoil loss, and enhancing soil microbial activity, but concerns over their water use has limited adoption in semi-arid regions [2–5]. Agricultural production in Mediterranean climates, classified by cool, wet winters, and hot, dry summers, is often reliant on irrigation inputs. Notably, Southern California and the San Joaquin Valley of California receive ~ 0–1 mm average monthly rainfall June through September [6], but produced a table grape crop valued at $2.3 billion USD in 2023 [7]. In general, table grape production involves intensive irrigation through harvest [8], as berry size is an important quality metric, unlike winegrape vineyards wherein devigoration is a common practice and cover crops are relatively well-studied [9–11]. This offers a unique opportunity to study a water-scarce agroecosystem with relatively high water demand, wherein rainfed cover crops are seeded adjacent to the commercial crop and terminated prior to the main growing season [12].

In semi-arid vineyards, cool-season cover crops are typically sown in interrow spaces between, but not beneath the vines and are terminated by May, when precipitation dwindles. In vine rows, vines are trellised, soils are maintained vegetation-free, and drip irrigation systems provide crop water requirements for the summer growing season. Under drip irrigation systems, grape roots largely remain shallow and are dominantly located along the drip line, rarely extending into interrow areas. Despite this separation, cover crop roots may extend into vine row soils, and their residues and shade can influence soil properties. Additionally, interrow soils may act as microbial reservoirs, seeding vine row soils with microorganisms, while cover crops themselves can alter the vineyard microclimate, indirectly shaping soil conditions [13, 14]. Understanding the extent and mechanisms of these interactions is essential for optimizing cover crop management in vineyards.

To explore these dynamics, we evaluated two cover crop treatments with distinct characteristics: Lacy phacelia (*Phacelia tanacetifolia*), a California native, broadleaf forb with a shallow taproot system and a low carbon-to-nitrogen ratio (C: N) in litter residues, and Cereal rye, *Secale cereale* cv. Merced, an introduced grass with fibrous root systems and higher residue C: N. These species differ significantly in their biomass production and residue chemistry, and root exudate profiles, leading to varied impacts on soil properties and microbial communities [4, 15, 16]. For instance, faster decomposition of the phacelia residues may enhance nutrient cycling, while rye’s fibrous roots and higher C: N ratio might contribute to longer-lasting soil organic matter [17]. These differential inputs likely mediate soil microbial community assembly, which is driven by biotic and abiotic factors [18, 19].

Microbial communities, essential drivers of soil health and nutrient cycling, dynamically respond to plant inputs such as root exudates and residues [20, 21]. However, there is currently a limited understanding of how different cover crops influence microbial communities, not only in the interrow during the winter and spring when cover crops are active, but also in the vine rows during the summer, when table grape vines are active, but the cover crops have long been terminated. Furthermore, temporal dynamics play a critical role, as the effects of cover crops on microbial communities tend to accumulate over time, with the most pronounced differentiation emerging after several years of consistent implementation [22]. Alternatively, initial impacts may be most pronounced owing to cover crops shaping the soil conditions during the reassembly of soil communities following major disturbances from vineyard establishment, soil grading, and berm generation [23].

Our study aims to address these knowledge gaps. We hypothesize that soil chemical and biological properties will experience more pronounced shifts in interrow soils under cover crop treatments, while vine row soils will show subtler changes over time. Furthermore, we hypothesize that the effects of cover crops on soil microbiomes will become increasingly distinct with longer management durations. This study further explores whether specific cover crops differentially influence the soil microbiome and soil health metrics across vineyard zones. To test these hypotheses, we conducted a three-year field study integrating microbial community profiling, soil physicochemical analyses, and plant-soil interaction assessments. This comprehensive approach enabled us to evaluate how cover crops modulate soil microbiomes, the temporal dynamics of these changes, and their associations with soil health metrics.

## Materials and methods

### Study site characteristics and soil sampling

In 2019 a table grape vineyard was established in Parlier, CA USA (36.5960049, -119.5119173) by grading an ~ 0.3-hectare plot and forming 0.9 m berms with 3.6 m spacings (Supplemental file 2: Figure S1). The vineyard soil is a is a Hanford soil series (Coarse-loamy, mixed, superactive, nonacid, thermic Typic Xerorthents), which is ubiquitous in the Eastern San Joaquin Valley of California. This region has a Mediterranean climate with cool wet winters and hot dry summers and mean annual precipitation of ~ 320 mm, well below the evapotranspiration demands of a vineyard during the growing season. Irrigation is supplied to vine rows via a metered drip irrigation system. The experiment design was a randomized complete block, with two grapevine rows per block and 12 vines per row within a treatment plot. In December 2019 cover crop treatments were seeded and included (i) Phacelia: a California native plant species mix comprised of California brome grass (*Bromus carinatus*), 40%; Blue wildrye (*Elymus glaucus*), 25%; Lacy-leaf Phacelia (*Phacelia tanacetifolia)*, 15%; Arroyo lupine (*Lupinus succulentus*), 10%; and Common yarrow (*Achillea millefolium*), 10%; (ii) Rye: an introduced plant species mix including Flecha tall fescue (*Lolium arundinaceum*), 35%; Berber orchardgrass (*Dactylis glomerata*), Merced Rye (*Secale cereale L.* ‘Merced’), 25%; Birdsfoot trefoil (*Lotus corniculatus*), 10%, and sour clover (*Trifolium facatum*), 10%, for comparison to (iii) Control: an unplanted control wherein interrow spaces were maintained bare using a glufosinate-ammonium (Lifeline^®^) herbicide. Seed mixes were broadcast seeded at a rate of ~ 33.6 kg ha^− 1^, then shallowly incorporated in the center ~ 1.8 m of the interrow space within each cover crop treatment plot. For each mix an early emerging species dominated (> 90% of planted emerged seedlings). Thus, this study focuses on comparisons of phacelia or rye treatments, not native and introduced species mixtures, and the unplanted control (Supplemental file 2: Figure S2). There were four blocks, and correspondingly four replicates per treatment or control condition, with each treatment plot covering an 11 m x 22 m area. Autumn King grapevines on Freedom variety rootstock were planted in May 2020. Cover crops emerged each year ~ January to February, were supplied supplemental irrigation in years 1 and 2 of the trial and were rainfed in year 3 to ensure dense establishment. Both the phacelia and rye cover crops self re-seeded, then dried and senesced around May in each year and were mowed, with residues left in place to serve as a mulch during the summer grapevine growing season. Additional details on the field trail establishment are available in a prior publication [4].

Field soil samples were collected in December 2019, serving as a baseline, then in the following April (peak cover crop biomass), June (vigorous grapevine growth and berry development), and September (berry ripening) of each year in 2020, 2021, and 2022. Soils were collected from the upper 0–20 cm soil depth using 5 cm diameter soil probes. Soil samples were collected separately from vine rows and adjacent interrow spaces and each soil sample consisting of a composite of three probes. In vine rows, soils were probed consistently, 0.6 m from vine trunks and 0.3 m from drip emitters in effort to minimize the variances imparted by grapevine roots and soil moisture and probes were spaced 3.7 m apart. Approximately, 1.8 m south of each vine row soil sampling location, a soil sample was collected in the center of the interrow. Thus, for each timepoint, a composited soil sample was collected from both the vine row (*n* = 4) and interrow locations (*n* = 4). All soil samples were mixed well, then sub-sampled for storage at -80 °C, -20 °C, or 4 °C for further analyses.

### Soil physicochemical analyses

Soil pH and gravimetric soil moisture were determined using standard techniques [24] using soil samples stored briefly (1–3 days) at 4 °C in airtight plastic bags. Briefly, soil pH was determined using a 1:5 soil to water ratio. Approximately, 20 g of dried soil samples were ground with mortar and pestle, sieved (840 μm mesh), and dried at 50 °C for 24 h prior to total carbon (TC) and total nitrogen (TN) analyses. A 0.25 g subsample of the 20 g sample was analyzed on a TruMac CN Leco analyzer (LECO Corp., St. Joseph, MI). The TC and TN percentages were used to calculate C: N ratios. Nitrate (NO_3_^−^) and ammonium (NH_4_^+^) were quantified using frozen soil (-20 °C) and 1 M KCl extracts processed on a LACHAT Quikchem 8500 Flow Injection Autoanalyzer (Hach Company, Loveland, CO, USA) [25, 26].

### Phospholipid fatty acid (PLFA) analysis

Soil samples were processed using a high throughput PLFA extraction procedure [27] with a few modifications. Briefly, soils stored at -80 °C were lyophilized, then 2 g (dry weight) subsamples were spiked with an internal standard. The standard was composed of 10,000 pmol of 19:0 phosphatidylcholine (Avanti Polar Lipids, Alabaster, AL, USA) dissolved in 1:1 chloroform: methanol. Soil PLFAs were purified using a solid phase extraction 96-well plate (Supelco 50 mg Discovery Plate, Millipore Sigma, Darmstadt, Germany) and esterified to generate fatty acid methyl esters (FAMEs). FAMEs were then dissolved in hexane (100 µL) and run on an Agilent 6890 gas chromatograph (Agilent Technologies, Wilmington, DE, USA) with peak naming software and a library of standards (PLFAD1) from MIDI Inc. (Newark, DE, USA) to analyze extracted FAMEs. With reference to the internal standard, total soil microbial biomass (SMB) in nmol PLFA extracted g^− 1^ dry soil was determined for each soil sample. Identified FAMEs were further categorized into broad microbial groups using Supplemental file 1. Based on the total SMB and the portions of FAME’s attributed to each group, we determined the relative abundances of arbuscular mycorrhizal (AMF) fungi, other fungi, Actinobacteria, other Gram-positive (GP) bacteria, Gram-negative (GN) bacteria, anaerobic bacteria, eukaryotes, or general FAME’s [28]. We also included fungi: bacteria (F: B), GN: GP, coptiotrophic: oligotrophic (Copio: Oligo), and saturated fatty acid: unsaturated fatty acid (Sat: Unsat) ratios as well as mono-unsaturated (MUFA) and poly-unsaturated fatty acids (PUFA) in our correlation analyses. Notably, F:B is sometimes used as a proxy for soil health, as fungi are often more disrupted by soil disturbance, but this concept has also faced criticism owing to oversimplification [29]. Several studies have associated GN: GP ratios as an indicator of soil C substrate quality, with increased GN: GP ratios when there is higher availability of labile soil C substrates [30]. Similarly, generalized life strategies of bacteria, growing quickly in nutrient rich conditions (copiotrophs) or slowly in nutrient poor conditions (oligotrophs) can be used to profile microbiome community characteristics and soil conditions. FAME signatures of GN organisms and Firmicutes were grouped as copiotrophs and all other GP FAMEs were grouped as oligotrophs [28]. In general, mono-unsaturated fatty acids (MUFA), as opposed to poly-unsaturated fatty acids (PUFA), are considered indicators of aerobic conditions with and high substrate availability, are associated with GN bacteria, and have been shown to discriminate land use [31]. We also included the predator: prey (Pred: Prey) ratio, calculated from the relative abundance of fatty acid biomarkers associated with non-fungal eukaryotes and prey organisms (most GP and GN bacteria). All FAME groups and ratios, including F: B, GN: GP, Copio: Oligo, Sat: Unsat, MUFA, PUFA, and Pred: Prey, were classified according to Supplemental file 1.

### Soil DNA extraction, preparation of 16 S rRNA gene and ITS amplicon libraries, and sequencing

Soils stored at -80 °C were lyophilized, then 0.25 g soil was used for DNA extraction using the Qiagen DNeasy PowerSoil kit following the manufacturer’s instructions. DNA quality was verified (260:280 *>* 1.75; 260:230 *>* 1.70) using a spectrophotometer (DS-11 FX+, DeNovix, Wilmington, DE, USA). Quantity of DNA per sample was determined using Invitrogen PicoGreen Quanti-IT dsDNA assay kits (ThermoFisher Scientific, Waltham, MA, USA). For each sample, 12.5–17.5 ng of DNA used to prepare triplicate reactions of a two-step PCR workflow with the first PCR step employing gene specific primers, followed by PCR clean-up with a HighPrep PCR reagent (MAGBIO Genomics, Inc, Gaithersburg MD, USA), and a second PCR step employing Nextera indexes for sample barcoding [32]. The first step included 0.5 µM each of primers 515f and 806r [33] to target bacterial 16 S rRNA gene sequences or primers gITS7 and ITS4 [34] to target ITS2 regions for fungal community profiling. Amplicon libraries were quantified using a Quant-IT PicoGreen ds-DNA specific reagent (Invitrogen), pooled at equal molality, gel purified (Qiaquick Gel Extraction Kit), and validated for accurate amplicon length on a bioanalyzer. For the negative control, a water-only control was included for each 96 well plate. Pooled libraries were sequenced at the UC Davis Genome Interrow on an Illumina MiSeq instrument in 2022 (2019–2021 samples) and 2023 (2022 samples). Temporal shifts in taxa based on year were validated by re-running select amplicon libraries from 2020, 2021, and 2022 on the same sequencing run in 2024 (Supplemental file 2: Table S2). The processing of 16 S rRNA gene and ITS genomic libraries was conducted using the sequence analysis tool within the IEG Data Management Pipeline (http://ieg3.rccc.ou.edu:8081/) following the DADA2 workflow [35]. After generating abundance tables of amplicon sequence variants (ASVs), a taxonomic annotation based on Silva taxonomic training (database v138.1) for 16 S rRNA and UNITE classifier v9.0 for ITS was performed. The quality step (filtering, denoising, and the removal of chimeras) on the abundance matrices was used to eliminate low prevalence sequences. ASVs assigned to phyla with a cumulative prevalence of only one sample across all associated ASVs were excluded prior to downstream analysis. Subsequently, ASVs identified as Chloroplast, Mitochondria, or Cyanobacteria were filtered out across all taxonomic ranks. This two-step filtering process removed 2,724 ASVs from the dataset. The final dataset contained 9,245,648 high-quality reads, with an average of 40,910 reads per sample prior to rarefaction. To standardize sequencing depth across samples, total 16 S rRNA gene reads were rarefied to an even depth of 36,205 reads per sample using the *rarefy_even_depth()* function in the R package ‘phyloseq’ v.1.48.0 [36]. A similar procedure was applied to the ITS dataset, resulting in 6,132 ASVs after quality filtering and rarefaction to 17,252 reads per sample.

### Data analysis

To identify the factors most influential in shaping the distribution of bacterial/archaeal and fungal sequences in this study, a variation partitioning analysis (VPA) [37] was performed using the ASV abundance table prior to rare ASV filtering [38]. To ensure inclusion of ASVs representative of treatment effects, a minimum prevalence threshold of 20% across samples within each treatment was applied, considering only ASVs detected at ≥ 0.01% relative abundance. This filtering was performed using the *core()* function from the microbiome R package (v.1.12.0) (Supplemental file 2: Figure S3). Non-metric multidimensional scaling (NMDS) using Bray–Curtis dissimilarities was employed to visualize treatment-driven differences in microbial community structure. Statistical comparisons were conducted via PERMANOVA with 10,000 permutations.

A principal component analysis (PCA) on the correlation matrix was used to identify soil variables most indicative of temporal trends under cover cropping (‘factoextra’ R package v.1.0.7 [39]). The selected variables were subsequently tested for treatment-level differences using linear models fitted over the full temporal dataset (baseline to Year 3). Random Forest classification models were trained (‘ranger’ R package v.0.16.0 [40]) to select the top 10 variables best differentiating treatments within interrow and vine row zones. To minimize early-stage variability, the analysis focused on data from Years 2 and 3, based on the PCA results (Figure S4). All variables were normalized using the min-max method [41]. A bootstrap resampling strategy with 1,000 iterations and treatment-stratified sampling was used. Variable importance was ranked by average Gini impurity across bootstraps, and treatment-level differences were tested using linear mixed-effects models (‘lme4’ R package v.1.1–35.3 [42]). Variables used in dbRDA were selected from the Random Forest rankings to retain only the most informative predictors.

Functional prediction analyses were conducted using the FAPROTAX [43] and FUNGuild [44] databases for bacteria and fungi, respectively, to assess microbial functions after three years of cover crop treatments. A correlation-based co-occurrence network analysis was performed to compare microbial community topologies in vine row and interrow soils to baseline networks. Only significant associations (*p* < 0.001) with correlation coefficients > 0.70 were retained. Network layout and aesthetics were generated in Gephi (v.0.9.2), with node sizes scaled by degree and colored by module affiliation.

All *p*-values were corrected for multiple testing using the false discovery rate (FDR) method [45]. Microbiome analyses were conducted using ‘dada2’ (v.1.32.0; [46]), ‘ampvis2’ (v.2.8.9; [47]), ‘microeco’ (v.1.8.1; [48]), and other dependencies in R v.4.4.0 [49]).

## Results

### Cover crops impact the structure of soil prokaryotes and fungal communities in vine rows and interrow spaces, which become more distinct with time

**Fig. 1:**
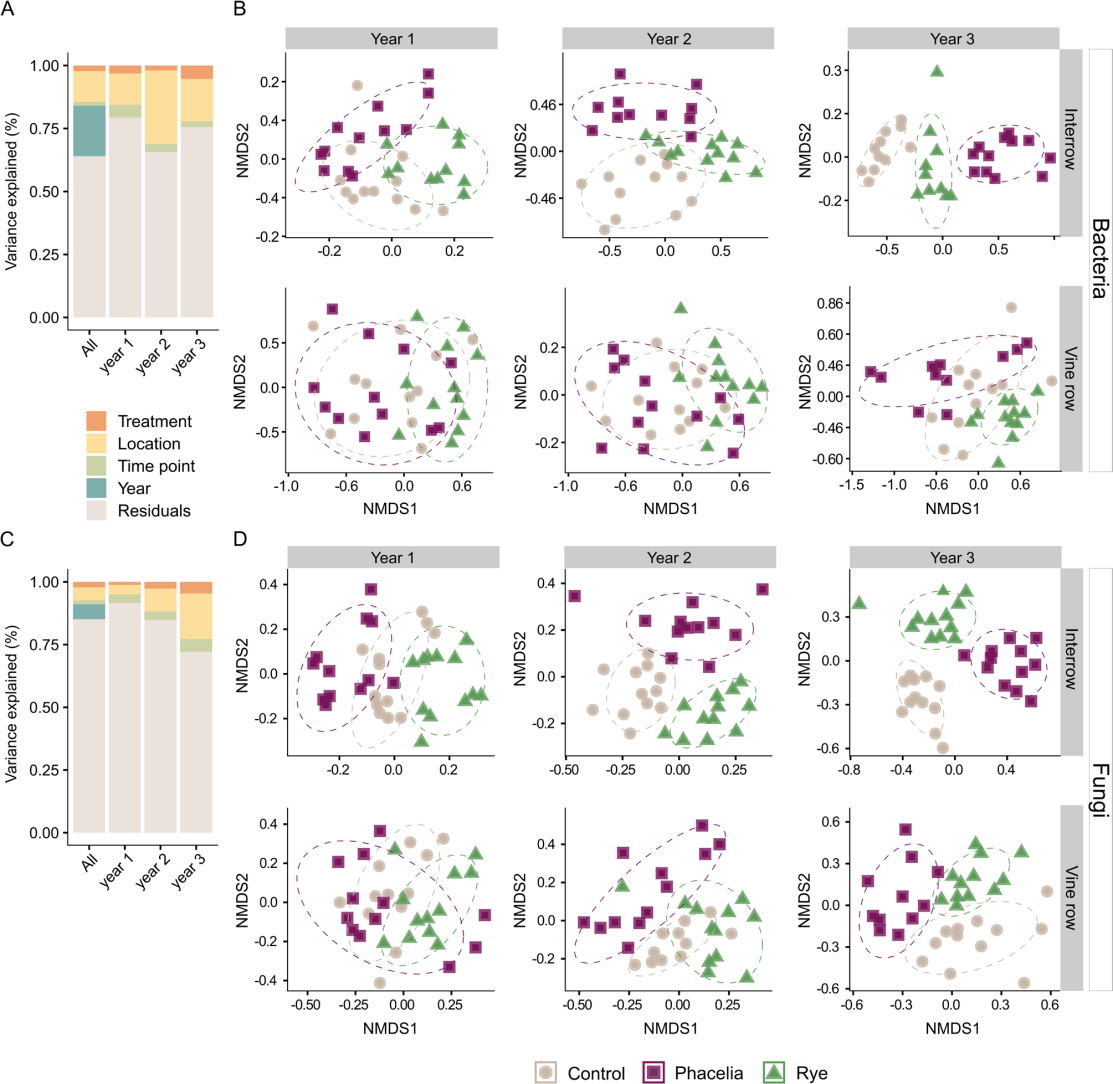
Temporal and spatial differentiation of bacterial and fungal communities under cover crop treatments. Variation partitioning analysis showing variance explained by treatment, location, time point, and year for bacterial (**A**) and fungal (**C**) communities based on the full ASV abundance table. NMDS ordinations of bacterial (**B**) and fungal (**D**) beta diversity were performed using core microbial community data (ASVs present at ≥ 0.01% relative abundance in ≥ 20% of samples) in interrow (top) and vine row (bottom) soils across time (years) and treatments: control (beige circles), phacelia (purple squares), and rye (green triangles)

Our VPA analysis revealed that among the influential factors in this study, time (year) explained the highest variation (20%) in the structure of prokaryotic communities, followed by location (12%), treatment (2%), and time point (1.5%) (Fig. 1A). However, for fungi, a similar explained variation was observed between year and location (~ 6%). Intra-annual analysis indicated erratic explained variation for bacteria. For fungi, the importance of each factor increased progressively each year, especially for location (Fig. 1C). Year 3 marked the highest contribution (5%) of the treatment factor for both domains. NMDS of core prokaryotic and fungal communities revealed significant impact of both factors on beta diversity (Fig. 1B, D). Notably, treatments yielded more distinct microbial communities when soils in the interrow (permanova; *p* < 0.001, Supplemental file 2: Table S3) were evaluated relative to soils from vine rows (Bacteria, *p* = 0.002; Fungi, *p* = 0.004). The highest differentiation between treatments was observed in the third year, mostly in the interrow. In the vine row, while both domains exhibited statistically significant differences in their communities, the fungal core communities were more distinct and less dispersed compared to the core prokaryotic communities.

### PLFA and soil chemistry metrics reflect stronger temporal shifts in vineyard soils under cover crop management, relative to an unplanted control

**Fig. 2:**
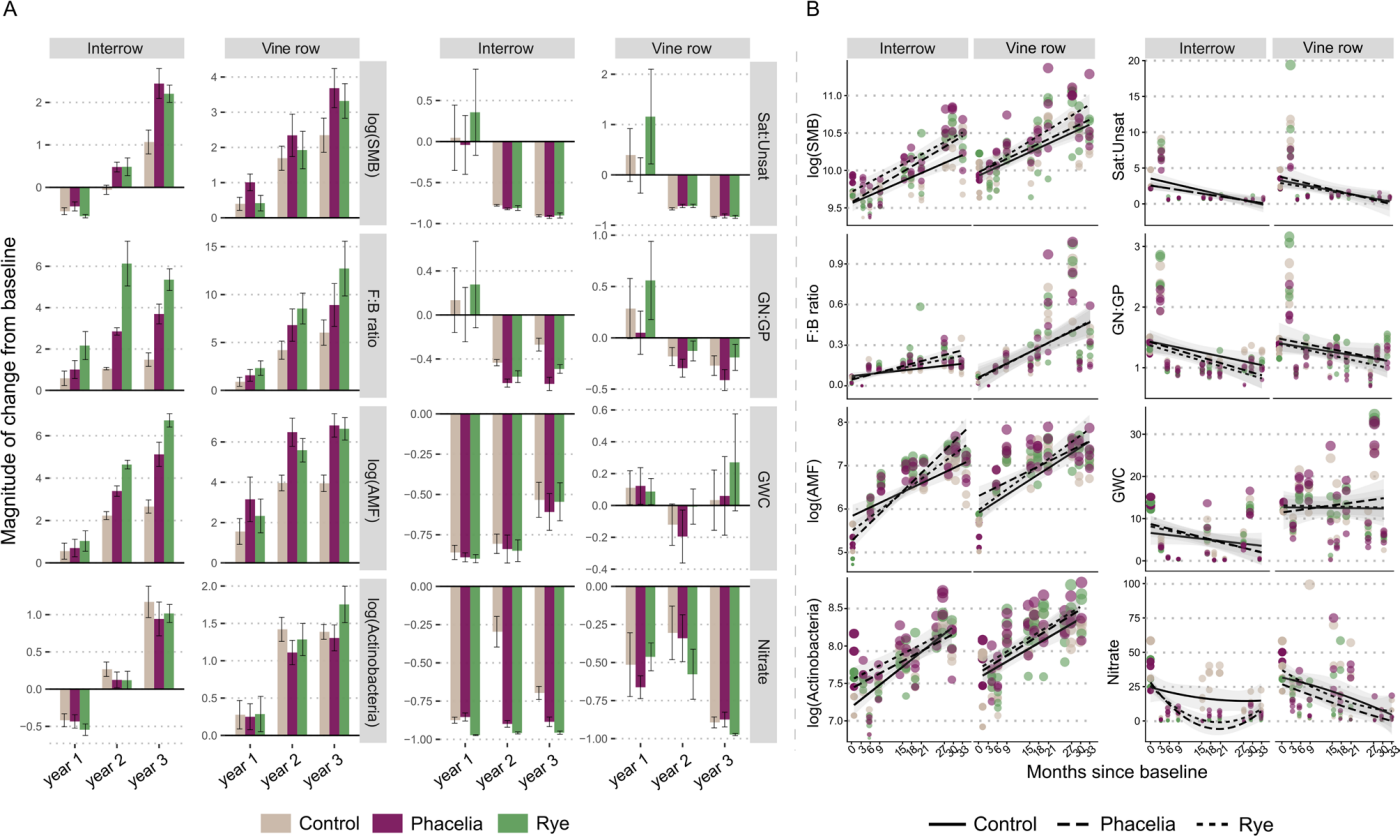
Temporal changes in soil microbial biomass and key soil metrics under cover crop treatments. (**A**) Magnitude of change from baseline for selected soil variables in interrow and vine row soils across years 1 to 3 under control, phacelia and rye treatments. (**B**) Linear regressions of selected variables over time across treatments: control (solid lines), native cover (dashed lines), and introduced cover (dotted lines). SMB – soil microbial biomass; F:B – fungi-to-bacteria ratio; AMF – arbuscular mycorrhizal fungi; Actinobacteria – bacterial group; GN: GP – ratio of Gram-negative to Gram-positive bacteria; Copio: Oligo – copiotrophic-to-oligotrophic ratio; Sat: Unsat – ratio of saturated-to-unsaturated fatty acids; GWC – gravimetric water content

The top 8 most important variables to distinguish temporal effects of cover crops were soil microbial biomass (SMB), F:B ratio, AMF, Actinobacteria, Sat: Unsat, GN: GP, GWC, and NO_3_^−^ (Supplemental file 2: Figure S4). Notably, across the three-year study, SMB nearly doubled in interrow and tripled in vine rows (Fig. 2A). The F: B ratios were higher in the cover crop treatment soils relative to the control soils starting from the second year, increasing by 6-fold in the rye treatment in both locations, and by 10-fold in the vine row soils by the 3rd year. The abundance of AMF was also higher in soil samples under cover crop management, in both locations, with rye yielding the greatest impact on AMF over time, reaching a 6-fold increase in the 3rd year. Linear models for each treatment between the selected variables and time since baseline revealed that many soil properties consistently shifted across years (Year 1 to Year 3), with some of these effects found consistent in both locations (Fig. 2B; Supplemental file 2: Table S4).

### Phacelia cover elicits stronger control over soil carbon, nitrogen, and bacterial/archaeal associations relative to rye

**Fig. 3:**
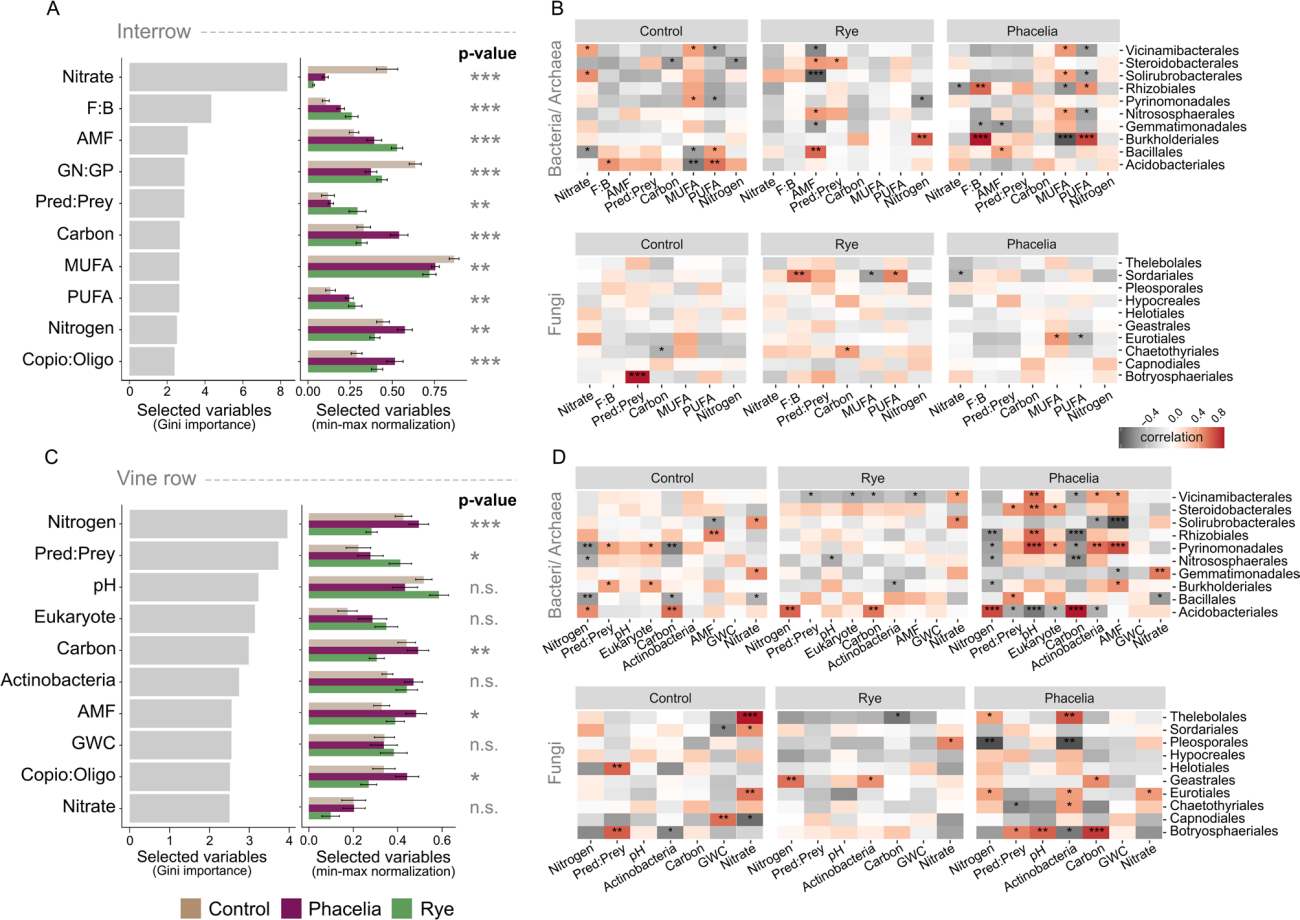
Random Forest-selected soil variables and their correlations with microbial taxa across treatments in interrow and vine row soils. (**A**) Top 10 soil variables distinguishing treatments in the interrow based on Gini importance and normalized values. (**B**) Heatmaps showing correlations between selected soil variables and bacterial and fungal taxa (Order level) across treatments (control, phacelia, rye) in the interrow. Microbial taxa were selected using Kruskal-Wallis tests (*p* < 0.05). Environmental variables were selected via Random Forest and tested for treatment effects using linear mixed-effect models. (**C**) Top 10 soil variables distinguishing treatments in the vine row. (**D**) Heatmaps showing correlations between selected soil variables and microbial taxa in the vine row. F: B – fungi-to-bacteria ratio; AMF – arbuscular mycorrhizal fungi; GN: GP – ratio of Gram-negative to Gram-positive bacteria; MUFA – monounsaturated fatty acids; PUFA – polyunsaturated fatty acids; Pred: Prey – predator-to-prey ratio; Copio: Oligo – copiotrophic-to-oligotrophic bacteria ratio; GWC – gravimetric water content. Statistical significance is indicated as *p < 0.05, **p < 0.01, and ***p < 0.001

Variable selection using Random Forest identified the 10 most sensitive variables for distinguishing treatments in both locations (Fig. 3A, C). Nitrate ranked highest in Gini importance in the interrow, with significantly higher levels in the control treatment than in cover crops (F = 57.54, *p* < 0.001). F: B ratio, AMF, Pred: Prey, and PUFA were key PLFA metrics in cover crop samples, with slightly higher values under rye. Total carbon and TN contents were significantly higher in phacelia treatment soils. Correlations between biotic and abiotic variables and prokaryotic ASVs (Order level) were stronger in phacelia, especially for bacteria (Fig. 3B, D). Specifically, *Burkholderiales* showed positive and strong correlations (*r* = 0.70, *p* < 0.001) with F: B ratio and PUFA, as well as strong negative correlations with MUFA. *Solirubrobacterales* exhibited negative correlations with AMF (*r* = -0.64) in the rye treatment interrow. Few significative correlations were observed between eukaryotes and the selected variables for distinguishing treatments in the interrow (Fig. 3B); *Botryosphaeriales* positively, strongly correlated with Pred: Prey (*r* = 0.77, *p* < 0.001). Regarding vine row, only 5 variables selected by the RF model were significantly different between treatments, and TN was the only soil variable with a highly significant difference (F = 10.82, *p* < 0.001) (Fig. 3C). Soil pH exhibited positive correlation (*r* = 0.60, *p* < 0.01) with ASVs from *Vicinamibacterales*,* Steroidobacterales*,* Rhizobiales*, and *Pyrinomonadales*, and strong negative correlation with *Acidobacterales* (*r* = -0.65, *p* < 0.001), which was positively associated with TC and TN. Relative to other abundant orders, in the vine row *Pyrinomonadales* had the strongest positive correlation with AMF. Regarding Fungi, *Botryosphaeriales* exhibited the highest number of significantly positive correlations, including Pred: Prey, pH and Actinobacteria. *Pleosporales* exhibited only negative and moderate correlations (*r* = -0.60, *p* < 0.01), especially with TN and TC.

### Phacelia and rye drive distinct beta diversity patterns across zones of vineyard soils

**Fig. 4:**
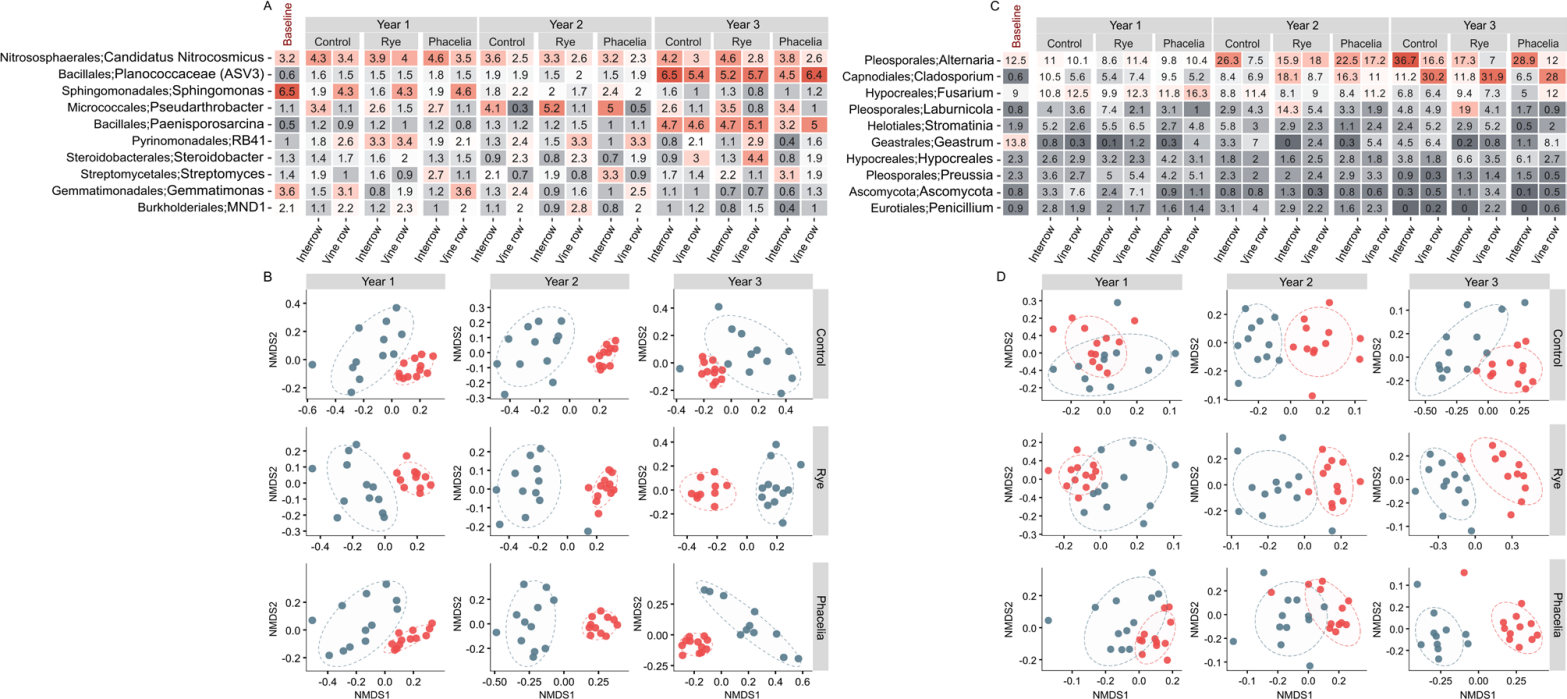
Spatial shifts in prokaryotic and fungal community composition across treatments and time. (**A**) Relative abundance of dominant bacterial and fungal orders over three years of cover crop management in interrow and vine row soils. (**B**, **C**) Non-metric multidimensional scaling (NMDS) ordinations showing beta diversity of bacterial (**B**) and fungal (**C**) communities under control, rye and phacelia treatments across years 1, 2, and 3. Statistical significance of treatment effects was assessed using Permanova

Overall, the interrow and vine row communities are becoming more dissimilar from one another with time, a trend which is intensified with interrow cover cropping (Fig. 4). Although the core communities were distinct in the first year (permanova, *p* < 0.01), the permutation test for homogeneity of multivariate dispersions showed dissimilar dispersion between communities under cover crop influence (permdisp, phacelia: F = 7.606, *p* = 0.018; rye: F = 7.305, *p* = 0.015), not being statistically significant (*p* < 0.05) for control. A more defined community structure profile was established in the second year, and the phacelia treatment promoted the greatest distinction in beta diversity across the locations compared to other treatments in the third year (permanova, R² = 0.304; *p* < 0.001). Finally, permdisp analysis revealed non-significant results for phacelia (F = 0.075; *p* = 0.783) and rye (F = 0.068; *p* = 0.793), suggesting a shift from stochastic processes predominating in the early stages of cover crop establishment to deterministic processes in subsequent assessments.

To assess temporal patterns in the soil microbiome and the influence of vineyard zones (interrow and vine row), we analyzed the relative abundance of bacterial and archaeal groups over three years of cool-season cover crop management. Baseline samples were included for comparison to evaluate changes attributable to cover crop treatments over time (Fig. 4A). *Sphingomonas* (*Sphingomonadales*) emerged as the most abundant genus before the experiment (6.5%), maintaining a relative abundance of approximately 4.5% in the vine row until the end of the first year. Subsequently, its proportion aligned more closely with the interrow community. Similarly, *Gemmatimonas* (*Gemmatimonadetes*) and MND1 (*Burkholderiales*) were initially more prevalent in the vine row compared to the interrow, a pattern that persisted into the second year.

In the interrow, *Pseudarthrobacter* (*Micrococcales*) consistently exhibited the highest relative abundance throughout the three years of the study, with proportions peaking in the second year, particularly under the rye and phacelia treatments. *Candidatus Nitrocosmicus* (*Nitrososphaerales*) demonstrated persistence from the baseline through the final year, maintaining abundances between 3.2% ~ 4.6% of the prokaryotic community in the interrow and 2.3% ~ 4.6% in the vine row, with minimal variation across treatments and consistent patterns over time. In contrast, *Planococcaceae* (ASV3) and *Paenisporosarcina*, belonging to the order *Bacillales*, were notable only in the third year, showing similar abundances across locations and treatments. *Streptomyces* (*Streptomycetales*) was significatively more abundant in the interrow with phacelia coverage (LDA, *p* = 0.003), maintaining higher relative abundance ​​than the other treatments throughout the 3 years of study (Supplemental file 2: Figure S5). Over time, the dynamics of the prokaryotic community structure revealed distinct formations between the vine row and interrow as early as the first year, with comparable dispersion and statistically significant differences across all treatments (permanova, *p* < 0.001) (Fig. 4B). By the third year, this differentiation became more pronounced, as evidenced by the increased distance between the centroids of multidimensional dispersion in samples from the rye (R² = 0.364; *p* < 0.001) and phacelia (R² = 0.363; *p* < 0.001) treatments, compared to the control (R² = 0.245; *p* < 0.001) (Supplemental file 2: Table S5).

At the study’s baseline, *Geastrum* (*Geastrales*), Alternaria (*Pleosporales*), and *Fusarium* (*Hypocreales*) were the most abundant fungal genera (Fig. 4C). In the first year, *Alternaria* was most prevalent in control areas, while *Fusarium* showed higher abundance under phacelia, with elevated values also observed in other treatments. By the second year, *Laburnicola* (*Pleosporales*) reached its peak abundance (14.3%) in the interrow under rye cover, and *Cladosporium* (*Capnodiales*) recorded its highest abundance (18.1%) in the interrow under rye cover. Cladosporium also displayed relatively high values in the interrow with phacelia (16.3%), contrasting with lower levels in the interrow of the control (8.4%). In the third year, *Alternaria* achieved its highest relative abundance (36.7%) in the interrow under the control treatment. *Cladosporium* exhibited significantly higher abundance in the vine row compared to the interrow, with consistent values across treatments, while *Fusarium* remained relatively stable across years in phacelia areas. Finally, *Laburnicola* had the highest relative abundance in the interrow under the rye treatment. The NMDS analysis highlighted a pronounced temporal effect of treatments on the beta diversity of fungal communities, emphasizing the evolving influence of management practices over time (Fig. 4D, Supplemental file 2: Table S6). Across all treatments, but especially in the control, a greater overlap in sample dispersion was observed between the interrow and vine row.

### Key soil variables and microbial functions shaped by cover crop treatments

**Fig. 5:**
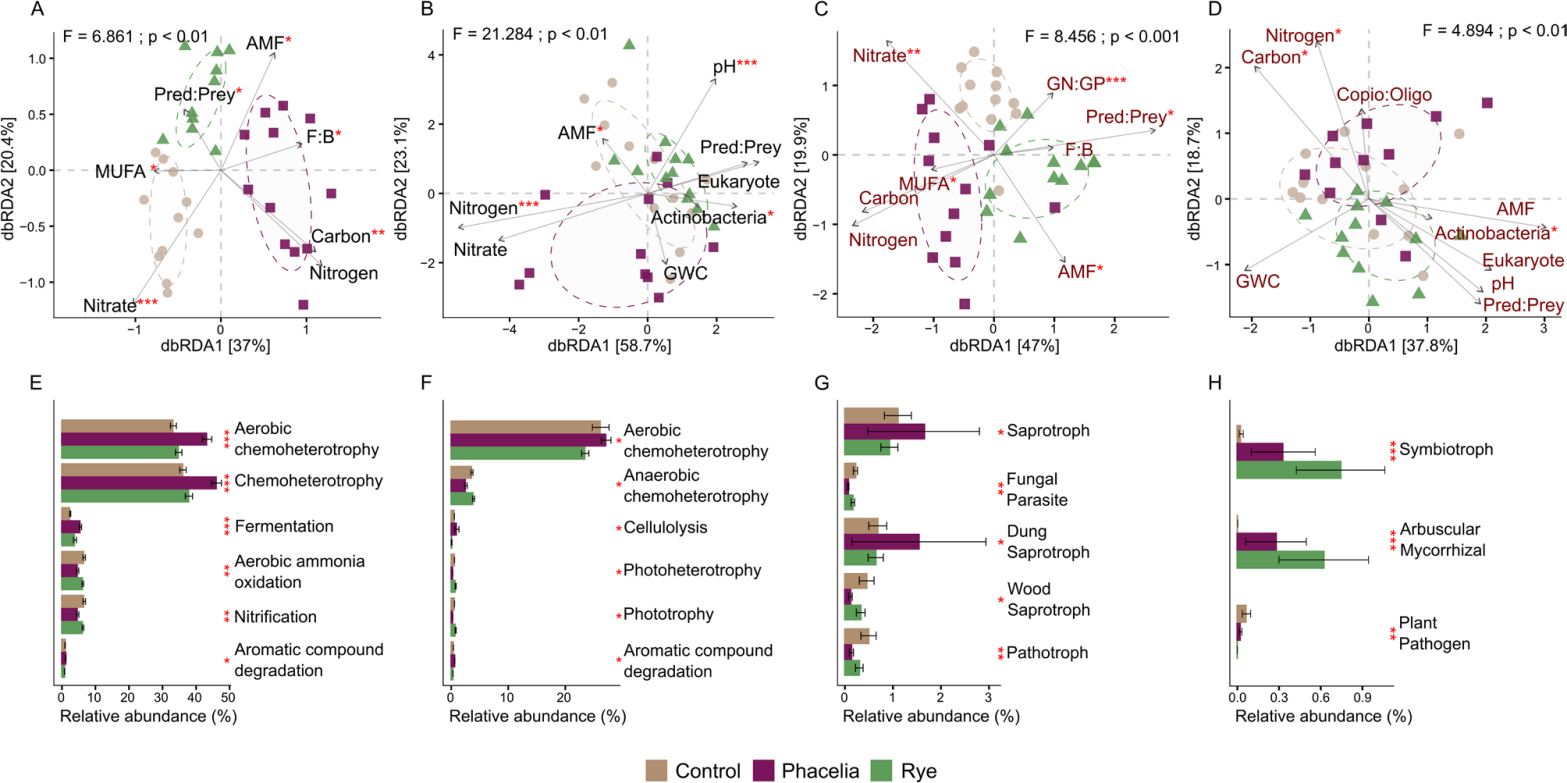
Relationships between microbial diversity, soil properties, and functional predictions under cover crop treatments. Distance-based redundancy analyses (dbRDA) showing relationships between microbial beta-diversity and selected soil variables for prokaryotic (**A**, **B**) and fungal (**C**, **D**) taxonomic profiles in interrow and vine row soils, respectively. Vectors represent the relative influence of key variables on community composition. Predicted functional profiles of microbial communities based on FAPROTAX and FUNGuild analyses, highlighting differences in metabolic and ecological functions across treatments in interrow (**E**, **G**) and vine row (**F**, **H**) soils. F: B, fungi-to-bacteria ratio; AMF, arbuscular mycorrhizal fungi; Pred: Prey, predator-to-prey ratio; MUFA, mono-unsaturated fatty acids; PUFA, poly-unsaturated fatty acids; GWC, gravimetric water content. Statistical significance is indicated as *p < 0.05, **p < 0.01, and ***p < 0.001

A strong association between the beta-diversity of prokaryotes and key soil variables was evident as revealed by dbRDA constrained ordinations (Fig. 5). The permutation test for dbRDA under the reduced model highlighted NO_3_^−^ and TC as the most influential variables driving the multivariate dispersion of prokaryotic communities in the interrow (Fig. 5A), acting as strong vectors separating the control-associated community from those under cover crop treatments. Similar trends were observed for fungal communities (Fig. 5C). The F: B ratio, MUFA, Pred: Prey, and AMF also played significant roles in shaping community structure. For the vine row, selected variables effectively distinguished prokaryotic communities across treatments, with TN and pH emerging as the most significant factors (*p* < 0.001) for bacteria (Fig. 5B). Regarding the fungal community, TN, TC, and Actinobacteria were moderately effective (*p* < 0.05) in explaining community structure changes (Fig. 5D).

Functional prediction analysis using FAPROTAX revealed that aerobic chemoheterotrophy, chemoheterotrophy, and fermentation were the most abundant predicted functions in the interrow under phacelia (*p* < 0.001). Aerobic ammonia oxidation and nitrification were more prevalent in rye and control compared to phacelia (*p* < 0.01). While treatment effects on predicted functions in the vine row were minimal, aerobic chemoheterotrophy remained the dominant function in this location (Fig. 5F). FUNGuild predictions revealed that symbiotic and saprotrophic fungal guilds were more prevalent in fungal communities influenced by cover crops. Symbiotrophic and AMF guilds (*p* < 0.001) were more abundant under cover crop treatments, with rye having a stronger effect in vine row soils (Fig. 5H). In parallel, the AMF PLFA biomarker vector in the fungal dbRDA plots (Fig. 5C and D) also pointed toward the rye treatment. The microbial analytical methods have distinct limitations, but each portrays AMF communities responding positively to cover cropping, enhancing the robustness of this conclusion. Conversely, pathotrophic and plant pathogen functions were significantly more abundant in the control treatment (*p* < 0.01) (Fig. 5G, H).

### Microbial network topologies highlight the role of cover crops in vineyard systems

**Fig. 6:**
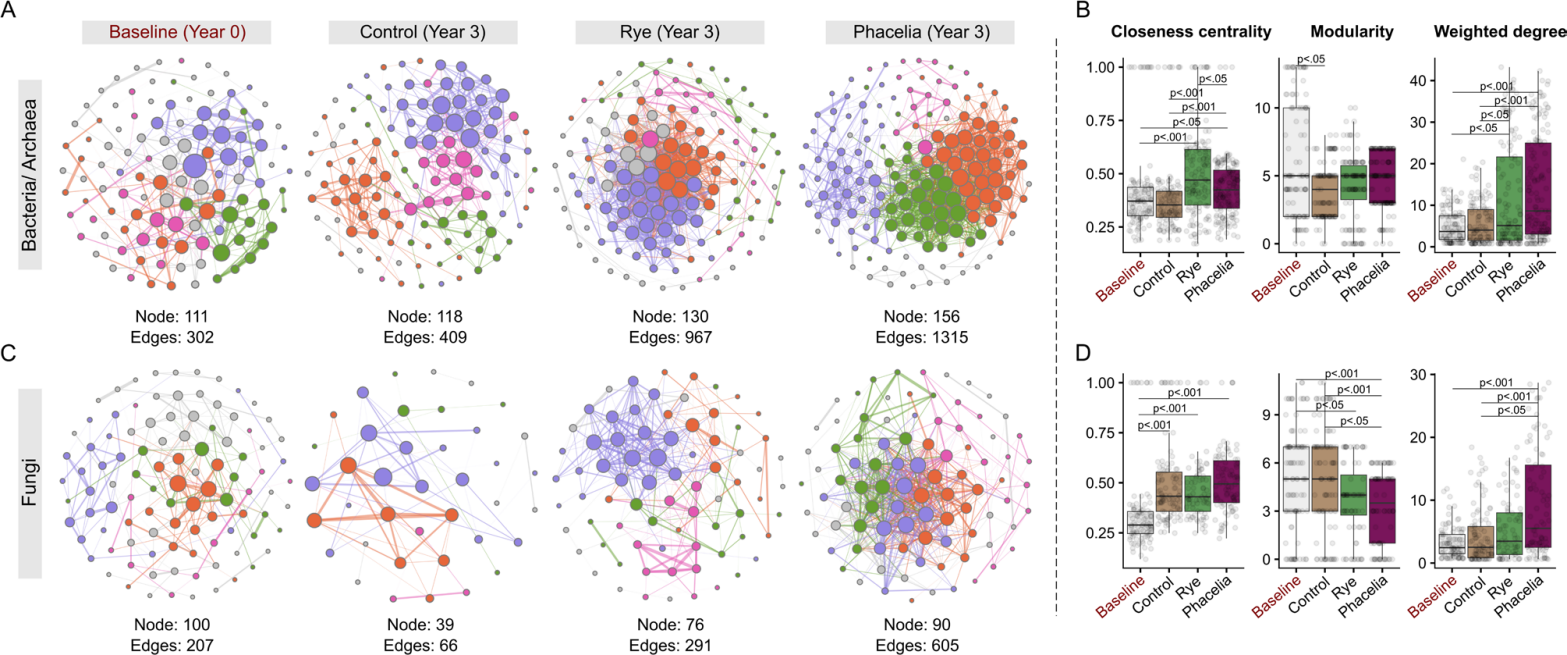
Microbial network and topology across cover crop treatments in vineyard soils. Microbial co-occurrence networks for bacterial (**A**) and fungal (**C**) communities at baseline (Year 0) and Year 3 for control, rye and phacelia treatments. Nodes represent microbial taxa, with edges denoting significant (*p* < 0.001) and strong (> 0.70) correlations. Colors represent modules (subcommunities). Topological metrics of microbial networks, including closeness centrality, modularity, and weighted degree, for bacterial (**B**) and fungal (**D**) communities across treatments and timepoints. Statistical comparisons highlight differences between treatments and baseline. Metrics include closeness centrality (network efficiency), modularity (network compartmentalization), and weighted degree (node connectivity). Statistical significance is indicated as *p < 0.05, **p < 0.01, and ***p < 0.001

By the third year of the study, both bacterial/archaeal and fungal networks exhibited distinct identities associated with the treatments, following a positive gradient of complexity: control < rye < phacelia (Fig. 6A, C). Topological features, including the number of nodes and edges, were numerically higher in the bacterial/archaeal networks under phacelia; however, no statistical differences in weighted degree were observed between cover crop treatments (Fig. 6B), although both differed significantly from the control and baseline. Compared to the baseline, bacterial/archaeal networks displayed lower overall complexity than fungal networks, with Fungi showing a greater number of nodes across treatments in the third year. Despite this, the lowest closeness centrality was recorded for the fungal networks, which mirrored the control in terms of modularity and weighted degree (Fig. 6D). This pattern suggests limited diversification of drivers or reduced functional performance within the fungal community in the absence of soil cover.

## Discussion

### Temporal and spatial drivers of microbial community differentiation

The results of this three-year study highlight the critical role of cover crops in shaping soil microbial dynamics during vineyard establishment. Profound effects of cover crops on both bacterial/archaeal and fungal populations were observed in the first year, but distinctions were markedly clearer in the third year, underscoring the importance of long-term evaluations to capture the cumulative impacts of cover cropping practices. By the third year, a clear differentiation among phacelia, rye, and control treatments emerged, revealing the influence of cover crop strategies on microbial community dynamics and soil properties. While the three-year duration of the study captured clear treatment effects, longer-term assessments would be valuable to evaluate the persistence and stability of these changes in microbial communities and soil health properties. These findings align with prior research emphasizing the capacity of cover crops to enhance soil health [50–52] and validate the hypothesis that temporal factors are key drivers of microbial community differentiation.

Minimal soil disturbance associated with perennial systems allows cover crop effects to accumulate with time compared to annual systems, where annual tillage can delay the detectable impacts of cover crops [53–55]. Spatial heterogeneity within perennial systems further accentuates this dynamic, as interrow and vine row soils offer contrasting ecological niches that shape microbial communities differently [56]. As expected, herein interrow soils, devoid of vine roots, exhibited stronger microbial responses to cover crop treatments, including higher microbial biomass and more pronounced differentiation between treatments. However, this trend was significant when soils collected across each growing season were pooled and evaluated and thereby most interrow soils revealed cover crop impacts in the absence of living cover crop roots. This observation supports the hypothesis that cover crops convey legacy impacts via residues and altered soil conditions, creating favorable conditions for microbial proliferation after their termination [4]. Complementary assessments of soil physical properties conducted during the same experiment showed that phacelia cover cropping significantly improved soil aggregate stability [4], corroborating the observed enhancements in microbial proliferation and soil health indicators reported in the present study. In addition to these legacy effects mediated by residues, the active growth phase of cool-season cover crops during winter likely contributed to soil modification through root exudation, nutrient uptake, and physical structuring [57]. Following plant senescence, decomposition of cover crop residues further enhanced soil carbon and nitrogen pools [10], while nitrate reductions and variations in gravimetric water content were observed across treatments. These cumulative changes, particularly pronounced in interrow soils where cover crops were established, likely created selective pressures that favored the differentiation of microbial communities by the third year [58].

Notably, vine row soils also displayed significant responses to cover crops, particularly in terms of microbial biomass and the F: B ratio. Interestingly, these metrics demonstrated a greater treatment effect in the vine row than in the interrow, which was not anticipated, particularly in early years of cover crop adoption. This finding highlights that vine row soils, while less distinct in terms of bacterial/archaeal and fungal community structure, still respond positively to cover crop management. Moreover, both arbuscular mycorrhizal fungi (AMF) and Actinobacteria responded to cover crop treatments, with AMF showing similar patterns of abundance across vineyard zones, while Actinobacteria exhibited consistent temporal changes, particularly under rye in the vine row. These patterns suggest that some microbial groups respond similarly across locations, whereas others exhibit more treatment or zone-specific dynamics [59].

Although this study was conducted during the non-bearing years of vineyard establishment, complementary data on vine vigor collected in the same experimental system revealed that phacelia cover cropping improved pruning weight and trunk diameter [4]. Ongoing work will examine table grape yield and quality to explore potential links between soil improvements and plant performance. These nuanced spatial dynamics partially confirm the hypothesis regarding spatial variation, revealing both expected and unexpected patterns in microbial responses to cover cropping.

Using predicted functional analyses, our results demonstrated a lower prevalence of fungal pathogens in cover-cropped plots compared to the control. This highlights the potential role of cover crops in mitigating disease pressure [60, 61], particularly under the rye treatment, which exhibited enhanced fungal biomass and AMF-associated symbiotic functions, a trend previously reported in the literature [62, 63]. While no significant difference in TN was observed between rye and the control, phacelia treatments exhibited consistently higher TN levels, particularly in the vine row. This treatment also demonstrated improvements in fungal biomass, indicating a dual role in enhancing soil health and indirectly managing pathogen dynamics. However, additional research on vine impacts is needed to address stress susceptibility under rye, which has been reported to reduce soil N and moisture availability in other systems [64].

### Complexity of microbial networks and shifts toward deterministic processes

The third year of the study revealed increasing complexity in microbial networks, with phacelia treatments consistently exhibiting the highest number of nodes and edges in both bacterial/archaeal and fungal networks. In contrast, the rye cover treatment displayed significant differentiation by year three, albeit with lower network complexity than phacelia. These findings align with previous research, indicating that native species may exert stronger ecological influences, potentially due to their evolutionary adaptation to regional conditions [61, 65]. The higher complexity observed in fungal networks compared to bacterial/archaeal networks is consistent with studies highlighting the pivotal role of fungal communities, particularly AMF, in soil aggregation and nutrient mobilization in agroecosystems [66].

Interestingly, while the phacelia treatment demonstrated the strongest impact on microbial network complexity, both cover crop treatments showed evidence of deterministic processes shaping microbial community assembly by the third year. This was evidenced by increased modularity and reduced stochastic variation in microbial networks, reflecting the influence of environmental pressures such as nutrient availability and water stress [67]. The presence of AMF and other functionally significant fungi under both treatments suggests that deterministic processes are fostering symbiotic relationships, enhancing nutrient cycling, and improving soil health [68]. These results indicate that specific traits of cover crops such as root architecture, litter chemistry, and exudate profiles can differentiate soil microbial communities and their associated ecological functions. Functional inference analyses based on FAPROTAX and FUNGuild suggested that cover cropping, particularly with phacelia, may promote microbial groups associated with key soil processes such as organic matter decomposition, nitrogen transformations, and symbiotic interactions. Although these predictions are based on taxonomic profiles and do not reflect direct measurements of functional activity, the observed shifts imply potential improvements in ecosystem services that are critical for soil fertility, resilience, and long-term plant health.

The observed shift toward deterministic processes, particularly under the rye treatment, emphasizes the role of environmental selection in microbial community assembly [67]. These processes are known to dominate under specific ecological pressures, such as those created by cover crop management in water-scarce regions. Importantly, the long-term application of cover crops appears critical in promoting microbial network complexity and resilience, highlighting their value in sustainable vineyard management.

### Cover crop differentiation of microbial communities are driven by nitrate and biotic soil metrics

Nitrate emerged as the most influential abiotic variable driving microbial community differentiation in interrow soils, with higher levels observed in control plots compared to cover-cropped treatments. This aligns with reduced nitrate availability in the rye cover, which may favor microbial taxa adapted to nutrient-limited conditions [69]. The treatment was also associated with reduced gravimetric water content, particularly in interrow soils. These patterns may reflect resource uptake by rye biomass during establishment and shifts in microbial community function potentially linked to changes in C: N dynamics. These patterns are consistent with previous findings that intensive resource competition or increased inorganic nitrogen availability can suppress microbial communities involved in biological nitrogen cycling, as demonstrated in vineyard soils under inorganic fertilization management [70].

In contrast, the Copio: Oligo ratio, representing the balance between fast-growing copiotrophs and slower-growing oligotrophs, was higher under phacelia treatments. This suggests a microbial community more reliant on readily available organic inputs, likely due to the lower C: N ratio in phacelia litter residues [4]. These findings illustrate the role of native species in providing a consistent nutrient supply, enhancing microbial growth, and bolstering ecological functions.

## Conclusions

This study underscores the role of cover crops in enhancing vineyard soil health by promoting microbial-mediated processes. The observed increases in microbial biomass, particularly fungal indicators such as F: B ratio and AMF, along with a greater prevalence of symbiotrophic and saprotrophic fungal guilds under cover crops, suggest enhanced microbial functioning in these systems. Additionally, the lower prevalence of fungal pathogens in cover-cropped soils suggests a potential disease suppression effect, emphasizing the multifaceted benefits of cover cropping [61]. These findings highlight the contribution of cover crops to shaping vineyard soil microbiomes and promoting multiple ecosystem services.

## Electronic supplementary material

Below is the link to the electronic supplementary material.

Supplementary Material 1

Supplementary Material 2

## Data Availability

The dataset supporting the conclusions of this article is available in the NCBI Sequence Read Archive repository, as part of the BioProject Accession PRJNA1207507. Fatty acid types included in each PLFA category are available in Supplemental file 1.

## Abbreviations

16 S rRNA: 16 S ribosomal RNA (rRNA)
ITS: Internal transcribed spacer
ASV: Amplicon sequence variant
PLFA: Phospholipid fatty acid
AMF: Arbuscular mycorrhizal fungi
Copio: Oligo: Copiotrophic-to-oligotrophic ratio
F: B: Fungi-to-bacteria ratio
GN: GP: Gram-negative to gram-positive ratio
MUFA: Monounsaturated fatty acids
PUFA: Polyunsaturated fatty acids
GWC: Gravimetric water content
Sat: Unsat: Saturated to unsaturated fatty acid ratio
SMB: Soil microbial biomass
Pred: Prey: Predator-to-prey ratio
Phacelia: Lacy phacelia (*Phacelia tanacetifolia*)
Rye: Cereal rye (*Secale cereale* cv. Merced)
