# Supplementary material for: Interrow cover crops in a semi-arid vineyard increase plant beneficial functional potential of the soil microbiome, both in vine rows and interrows, a benefit that increases with cover crop duration
Source: Environ Microbiome. 2025 Jun 11;20:66. doi: 10.1186/s40793-025-00726-1 (PMC12160431; doi:10.1186/s40793-025-00726-1)
Supplement: Supplementary file 2 — Supplementary Material 2 [file 40793_2025_726_MOESM2_ESM.docx]

Supplementary information for:

**Interrow cover crops in a semi-arid vineyard increase plant beneficial functional potential of the soil microbiome, both in vine rows and interrows, a benefit that increases with cover crop duration**

Fernando Igne Rocha ^1^, Jean Carlos Rodriguez-Ramos ^1^, Margaret Fernando ^1^, Lauren Hale ^1^

1. USDA, Agricultural Research Service, San Joaquin Valley Agricultural Sciences Center, 9611 South Riverbend Avenue, Parlier CA 93648-9757

*** Corresponding Author:** Lauren Hale (lauren.hale@usda.gov)

Supplemental information Text

**Soil variable selection for temporal effects**

A principal component analysis (PCA) was performed on the correlation matrix to identify soil health variables most indicative of the temporal effects of cover crop treatments relative to the control (bare soil) over the course of the experiment The data was standardized using *(xi – mean(x))/sd(x),* where *mean(x)* is the mean of x values, and *sd(x)* is the standard deviation (SD). The total contribution of a given variable on principal component axes was estimated. For example, the observed contributions of a variable on the first principal component, say PC1, was calculated with the formula: *[(C1 * Eig1) / (Eig1)*, where C1 are the contributions of the variable to PC1, and Eig1 the eigenvalues of PC1, respectively. The expected average contribution of a variable to PC1 is: *[(number of variables * Eig1)] / (Eig1).* In this study, the expected value was 1/length(variables) = 1/12 = 8.3%. In our results, variables with a contribution larger than this cutoff were considered as important in contributing to associated components. The visualization of the PCA was created with the *PCA()* function of the ‘FactoMiner’ R package v.2.11 (Husson et al., 2017).

Supplemental Figures


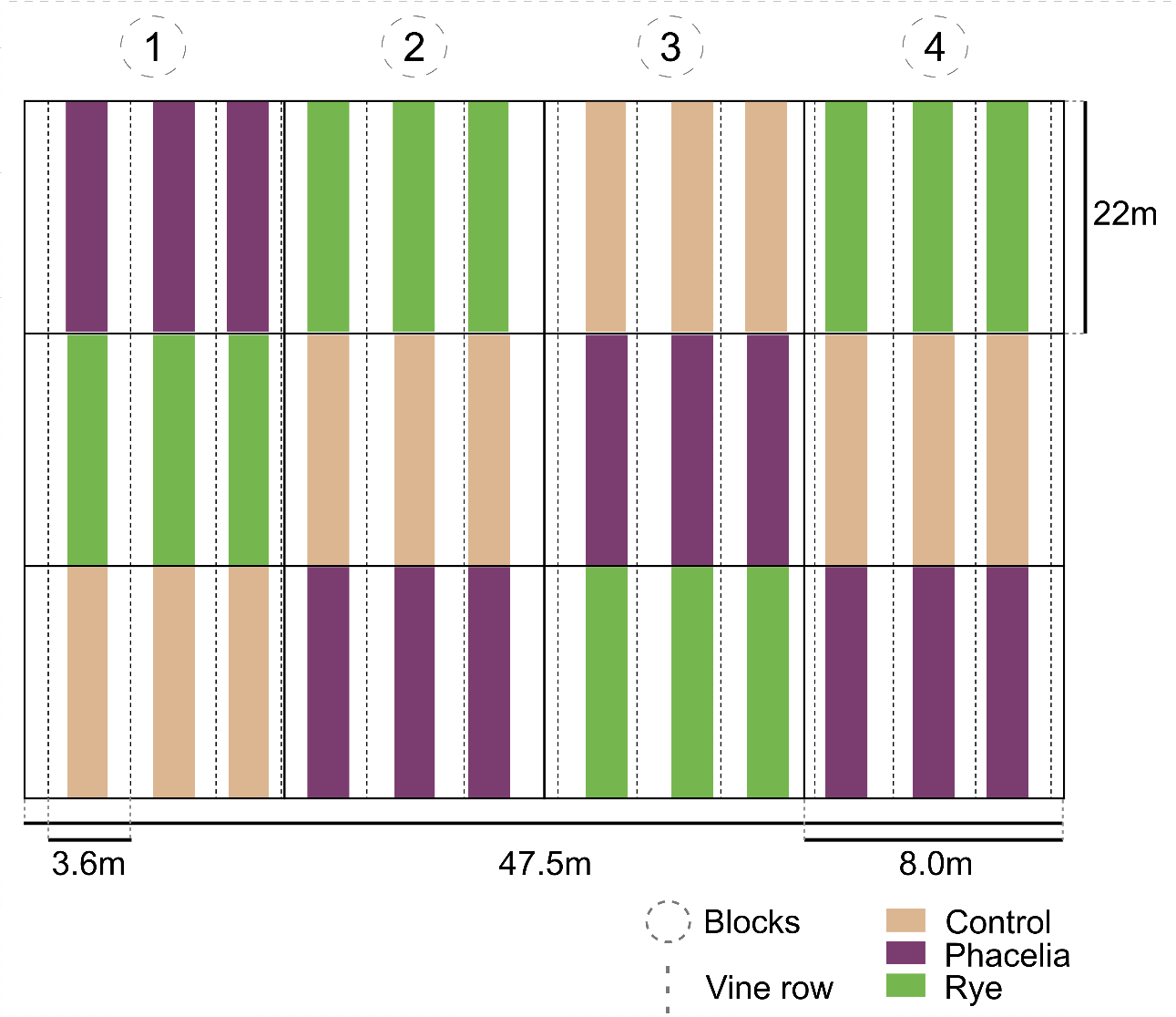


**Figure S1. Experimental design of the vineyard cover crop study.** Schematic representation of the experimental layout in a table grape vineyard, illustrating the spatial arrangement of cover crop treatments (Phacelia, Rye, and Control) in interrow zones. The experiment was structured in four replicated blocks (1–4), with alternating vine row and interrow sections. Each block consists of randomly assigned cover crop treatments applied in interrow spaces, while vine rows remained consistent across treatments. Dimensions of the experimental area are indicated for reference.


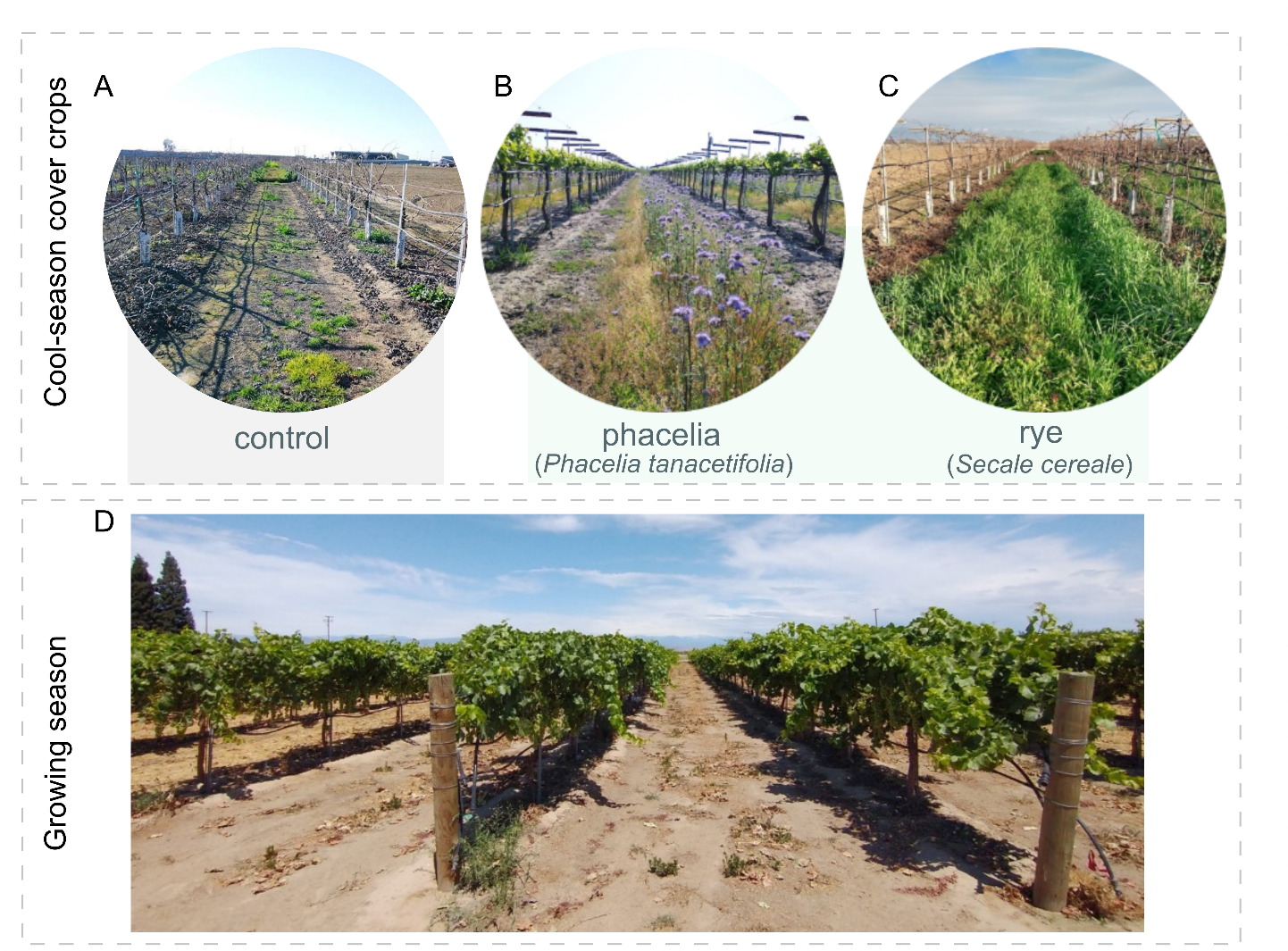


**Figure S2. Seasonal dynamics of cover crops in a table grape vineyard.** Representative images of the experimental cover crop treatments during the cool-season (A–C) and vineyard growth stage in summer (D). (A) Control treatment (bare soil), (B) interrow cover crop with Phacelia tanacetifolia, and (C) interrow cover crop with Secale cereale (rye). (D) Vineyard during the summer growing season, after cover crops have senesced, leaving residues on the soil surface.


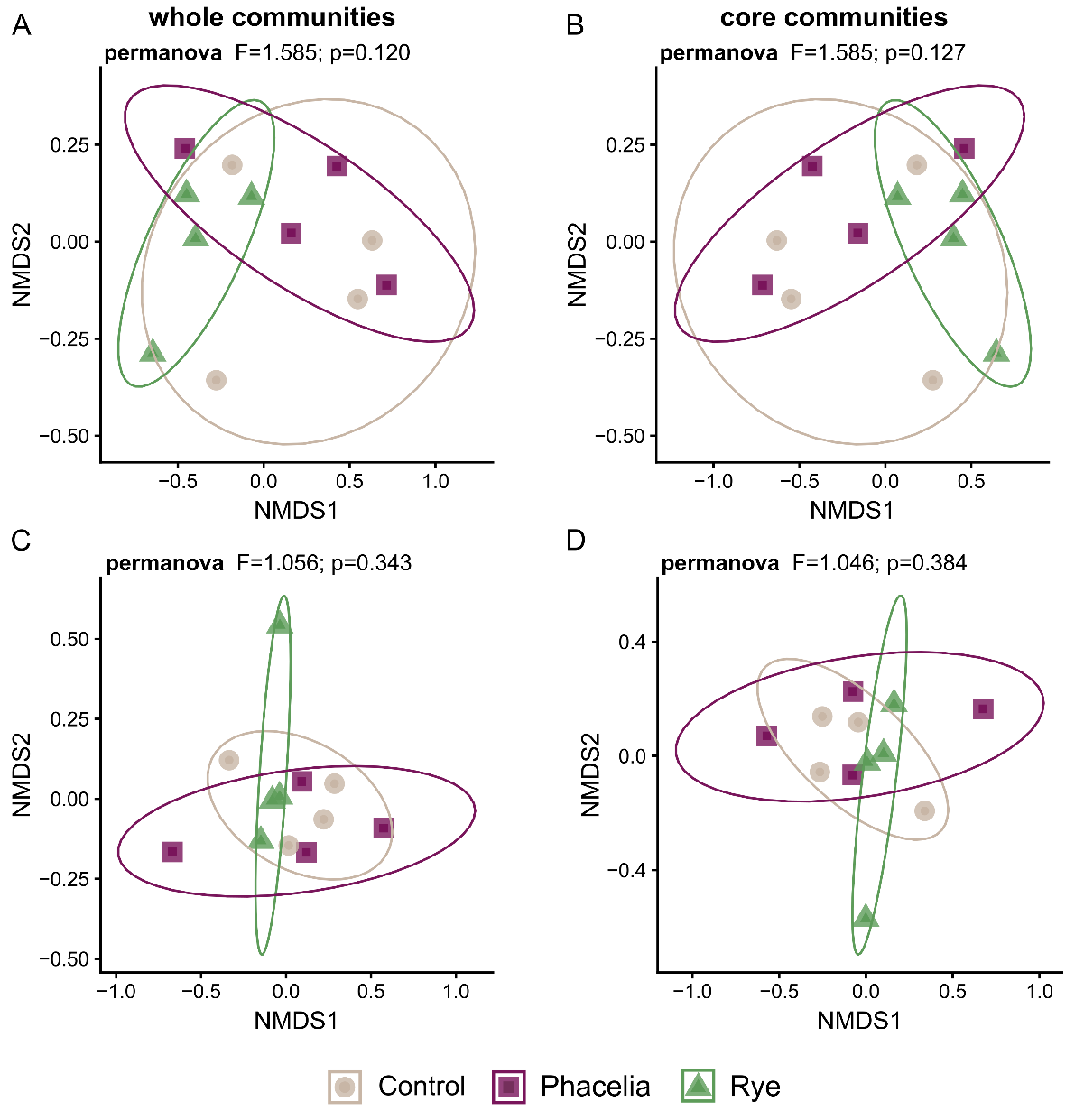


**Figure S3. Beta diversity of baseline samples before and after application of the core function.** Sample scatter based on NMDS ordinations of prokaryotic (16S rRNA; A-B) and eukaryotic (ITS; C-D) community structures. The core microbial communities were defined by ASVs detected at ≥ 0.01% relative abundance in ≥ 20% of samples. Permanova tests (p > .05) confirmed that application of the core filtering did not artificially introduce distinctions among microbial communities across treatments.


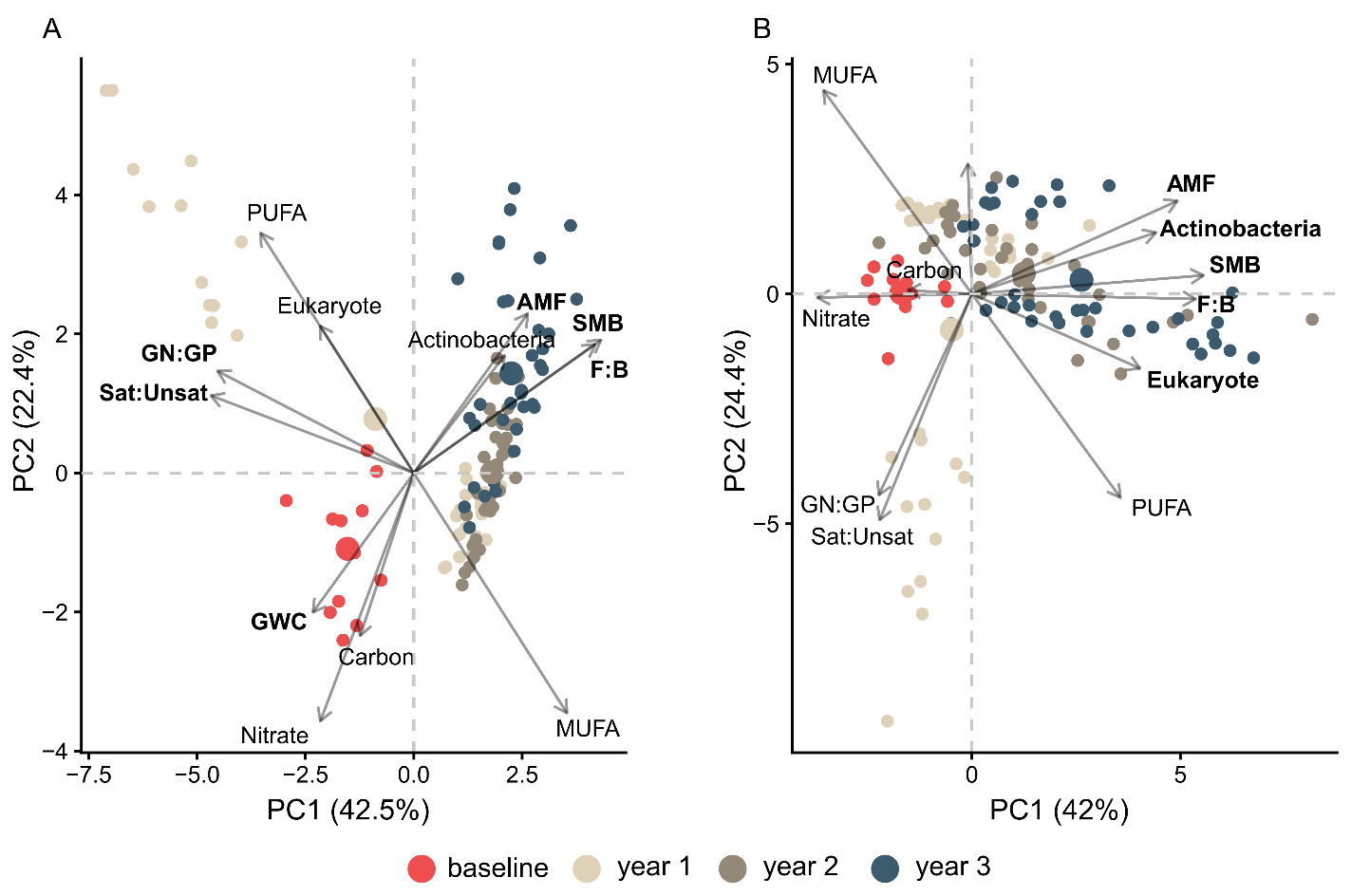


**Figure S4. Temporal differentiation of soil health variables in interrow and vine row soils based on PCA.** PCA results showing temporal changes in soil health variables under different treatments. (A) Interrow and (B) Vine row. AMF (arbuscular mycorrhizal fungi), F:B (fungi:bacteria ratio), GN:GP (Gram-negative:Gram-positive bacteria), PUFA (polyunsaturated fatty acids), MUFA (monounsaturated fatty acids), GWC (gravimetric water content), Sat:Unsat (saturated to unsaturated fatty acid ratio), SMB (soil microbial biomass), Carbon, and Nitrate.


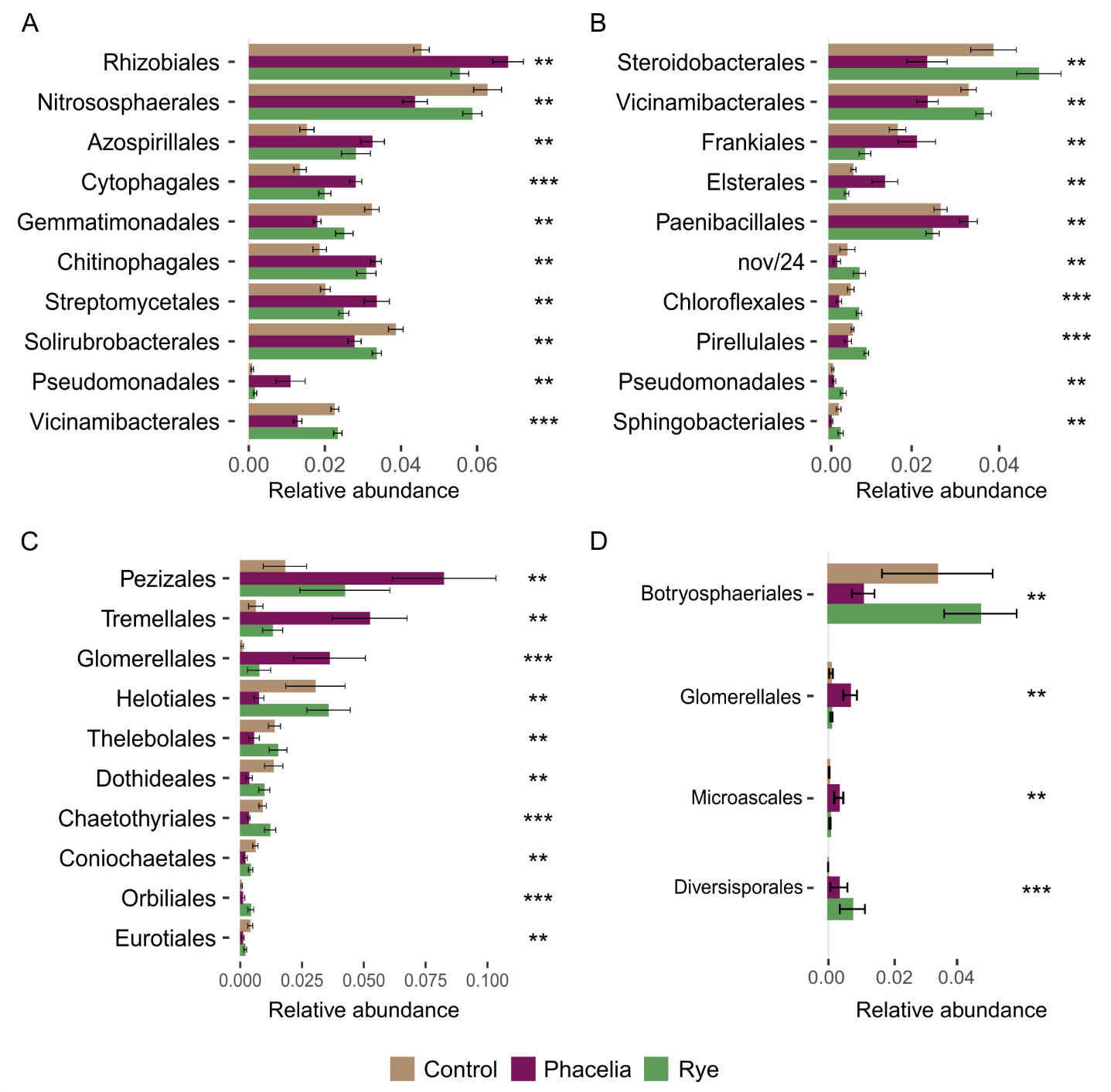


**Figure S5.** Differential abundance analysis of ASVs belonging to bacterial and fungal orders among treatments in Year 3 using linear discriminant analysis effect size (LDA-LEfSe). A-B, prokaryotes (16S rRNA), and C-D, eukaryotes (ITS).

Supplementary Tables

**Table S2.** Relative abundance of *Bacillales* groups in soil samples across years to evaluate potential biases from changes in 16S rRNA amplicon sequencing protocols.

| *Bacillales* groups | Year 1 | | Year 2 | | Year 3 | |
| --- | --- | --- | --- | --- | --- | --- |
|  | Phacelia | | Rye | | Phacelia | |
|  | Interrow | Interrow  (re-run) | Vine row | Vine row  (re-run) | Interrow | Interrow  (re-run) |
| *Planococcaceae* (ASV3) | 1.8 | **3.3** | 2.0 | **3.3** | 6.4 | **6.1** |
| *Paenisporosarcina* spp. | 1.2 | **2.7** | 1.5 | **2.6** | 3.2 | **3.6** |

**Table S3.** Permanova results comparing differences in bacterial and fungal community structure between treatments evaluated in both locations (vine row and interrow) during the experimental period.

| Domain | Location | Year 1 | | | Year 2 | | | Year 3 | |
| --- | --- | --- | --- | --- | --- | --- | --- | --- | --- |
|  |  | R^2^ adjusted | p-value | R^2^ adjusted | | p-value | R^2^ adjusted | | p-value |
| Bacteria | Interrow | 0.167 | p < 0.001 | 0.169 | | p < 0.001 | 0.257 | | p < 0.001 |
|  | Vine row | 0.141 | p = 0.002 | 0.135 | | p < 0.001 | 0.154 | | p = 0.002 |
| Fungi | Interrow | 0.121 | p < 0.001 | 0.133 | | p = 0.002 | 0.238 | | p < 0.001 |
|  | Vine row | 0.098 | p = 0.004 | 0.097 | | p = 0.002 | 0.098 | | p = 0.019 |

**Table S4.** Model performance metrics (R² adjusted and p-values) for the selected soil health variables based on linear regressions assessing changes over time (from baseline to Year 3) under different cover crop treatments and soil locations (interrow and vine row).

| Selected variables | | R^2^ adjusted | | p-value | |
| --- | --- | --- | --- | --- | --- |
|  |  | Interrow | Vine row | Interrow | Vine row |
| SMB | Control | 0.511 | 0.358 | < 0.001 | < 0.001 |
|  | Phacelia | 0.672 | 0.441 | < 0.001 | < 0.001 |
|  | Rye | 0.835 | 0.479 | < 0.001 | < 0.001 |
| F:B ratio | Control | 0.159 | 0.291 | 0.009 | < 0.001 |
|  | Phacelia | 0.388 | 0.212 | < 0.001 | 0.002 |
|  | Rye | 0.176 | 0.269 | 0.006 | < 0.001 |
| AMF | Control | 0.388 | 0.269 | < 0.001 | < 0.001 |
|  | Phacelia | 0.607 | 0.271 | < 0.001 | < 0.001 |
|  | Rye | 0.781 | 0.438 | < 0.001 | < 0.001 |
| Actinobacteria | Control | 0.600 | 0.440 | < 0.001 | < 0.001 |
|  | Phacelia | 0.477 | 0.424 | < 0.001 | < 0.001 |
|  | Rye | 0.709 | 0.461 | < 0.001 | < 0.001 |
| Sat:Unsat | Control | 0.241 | 0.231 | < 0.001 | < 0.001 |
|  | Phacelia | 0.248 | 0.214 | < 0.001 | 0.002 |
|  | Rye | 0.238 | 0.191 | < 0.001 | 0.004 |
| GN:GP | Control | 0.088 | 0.118 | 0.043 | 0.022 |
|  | Phacelia | 0.250 | 0.131 | < 0.001 | 0.017 |
|  | Rye | 0.166 | 0.123 | 0.007 | 0.020 |
| GWC | Control | 0.087 | n.s. | 0.044 | n.s. |
|  | Phacelia | n.s. | n.s. | n.s. | n.s. |
|  | Rye | 0.110 | n.s. | 0.026 | n.s. |
| Nitrate | Control | n.s. | n.s. | n.s. | n.s. |
|  | Phacelia | n.s. | n.s. | n.s. | n.s. |
|  | Rye | 0.130 | 0.247 | 0.017 | < 0.001 |

AMF (arbuscular mycorrhizal fungi), F:B (fungi:bacteria ratio), GN:GP (Gram-negative:Gram-positive bacteria), PUFA (polyunsaturated fatty acids), MUFA (monounsaturated fatty acids), GWC (gravimetric water content), Sat:Unsat (saturated to unsaturated fatty acid ratio), SMB (soil microbial biomass), Carbon, and Nitrate.

**Table S5.** Permanova outputs comparing bacterial community structure differences between vine row and interrow across treatments and years.

| Treatments | Year 1 | | | Year 2 | | | Year 3 | | |
| --- | --- | --- | --- | --- | --- | --- | --- | --- | --- |
|  | R^2^ adjusted | F | p-value | R^2^ adjusted | F | p-value | R^2^ adjusted | F | p-value |
| Control | 0.234 | 6.725 | 0.001 | 0.346 | 11.651 | 0.001 | 0.245 | 6.847 | 0.001 |
| Phacelia | 0.248 | 7.275 | 0.001 | 0.387 | 13.301 | 0.001 | 0.363 | 11.994 | 0.001 |
| Rye | 0.246 | 6.876 | 0.001 | 0.384 | 13.740 | 0.001 | 0.364 | 11.447 | 0.001 |

**Table S6.** Permanova outputs comparing fungal community structure differences between vine row and interrow across treatments and years.

| Treatments | Year 1 | | | Year 2 | | | Year 3 | | |
| --- | --- | --- | --- | --- | --- | --- | --- | --- | --- |
|  | R^2^ adjusted | F | p-value | R^2^ adjusted | F | p-value | R^2^ adjusted | F | p-value |
| Control | 0.099 | 2.419 | 0.003 | 0.229 | 6.239 | 0.001 | 0.239 | 6.916 | 0.001 |
| Phacelia | 0.122 | 3.076 | 0.001 | 0.141 | 3.638 | 0.001 | 0.304 | 9.211 | 0.001 |
| Rye | 0.145 | 3.736 | 0.001 | 0.190 | 5.183 | 0.001 | 0.247 | 7.251 | 0.001 |
